# Insights into the catalytic mechanism of formate dehydrogenases from different microbial sources

**DOI:** 10.1111/febs.70477

**Published:** 2026-03-05

**Authors:** Laura Legnani, Marco Gargiulo, Elia Lio, Enrico Mario Alessandro Fassi, Giovanni Grazioso, Maria Assunta Chiacchio, Barış Binay, Stefania Brocca, Francesco Secundo

**Affiliations:** ^1^ Department of Biotechnology and Biosciences University of Milano‐Bicocca Milan Italy; ^2^ Istituto di Scienze e Tecnologie Chimiche “Giulio Natta” CNR Milan Italy; ^3^ Dipartimento di Scienze Farmaceutiche University of Milan Milan Italy; ^4^ Dipartimento di Scienze del Farmaco e della Salute University of Catania Catania Italy; ^5^ Department of Bioengineering Gebze Technical University, Faculty of Engineering Kocaeli Türkiye; ^6^ BAUZYME Biotechnology Co. Gebze Technical University Technopark Region Kocaeli Türkiye

**Keywords:** FDH reaction mechanism, formate oxidation, hydride transfer, NAD^+^ reduction, QM/MM calculations

## Abstract

Four formate dehydrogenases (FDHs) from *Pseudomonas sp*. 101, *Myceliophthora thermophila*, *Chaetomium thermophilum*, and *Ogataea parapolymorpha* were recombinantly produced, purified, and characterized to investigate their catalytic properties and reaction mechanisms. The enzymes were studied for their ability to oxidize formate to carbon dioxide (CO_2_) coupled with NAD^+^ reduction. In contrast, their CO_2_ reduction activity was undetectable under the tested conditions. Oxidative reactions revealed significant differences in catalytic efficiency and substrate specificity, prompting further investigation through molecular dynamics (MD) simulations and quantum mechanics/molecular mechanics (QM/MM) ONIOM calculations. Structural models were derived from high‐resolution structural data available for enzymes from *Pseudomonas sp*. 101 (*pse*FDH) and *Chaetomium thermophilum* (*ct*FDH) and extended to all four variants. Comparative analyses of the transition states revealed distinct interaction patterns within the active sites, allowing us to discriminate between high‐ and low‐performing catalysts, in full agreement with the experimental *k*_*cat*_ values. These findings provide a mechanistic rationale for the observed disparities in catalytic performance and offer structural insights into the determinants of FDH activity. Notably, *ct*FDH emerged as a potential candidate for the development of CO_2_‐reducing reactions, with QM/MM data guiding the rational design of transition‐state stabilizing mutations.

## Graphical abstract

This integrated study combines experimental enzyme kinetics with QM/MM simulations to map the catalytic mechanisms of four formate dehydrogenases at the atomic level. This approach reveals the key determinants of catalytic efficiency and guides the rational design of biocatalysts for effective CO_2_ reduction—a crucial step towards sustainable biotechnology.

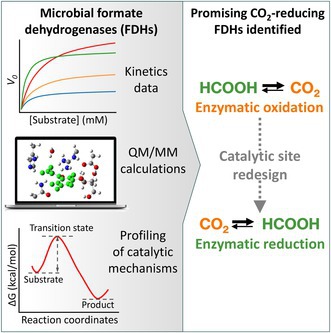

**Abbreviations**
APE: Absolute percentage errors for direct reaction
*ct*FDH: *Chaetomium thermophilum* FDH
DIC: Dissolved inorganic carbon
Exp *k*_*cat*_
: Experimental *k*_*cat*_
FDH: Formate dehydrogenase
IMAC: Immobilized metal ion affinity chromatography
MD: Molecular dynamics
*mt*FDH: *Myceliophthora thermophila* FDH
*op*FDH: *Ogataea parapolymorpha* FDH
*pse*FDH: *Pseudomonas sp*. 101 FDH
QM/MM: Quantum mechanics/molecular mechanics
RMSD: Root mean square deviation
SEC: Size exclusion chromatography
TS: Transition state
TS_*ct*
: *ct*FDH transition state for direct reaction
TS_*ct*‐rev: *ct*FDH transition state for reverse reaction
TS_*mt*
: *mt*FDH transition state for direct reaction
TS_*mt*‐rev: *mt*FDH transition state for reverse reaction
TS_*op*
: *op*FDH transition state for direct reaction
TS_*op*‐rev: *op*FDH transition state for reverse reaction
TS_*pse*
: *pse*FDH transition state for direct reaction
TS_*pse*‐rev: *pse*FDH transition state for reverse reaction
*ΔG* ^*‡*^
: Activation‐free energy
*ΔG* ^*‡*^_*exp*_
: Experimental free energy barriers, derived from *k*_*cat*_ values
*ΔG* ^*‡*^_*QM*_
: Activation free energy computed via QM/MM simulations

## Introduction

Formate dehydrogenases (FDHs; EC1.2.1.2) are oxidoreductive enzymes widespread in all living organisms. They catalyze the oxidation of formate ions to carbon dioxide (CO_2_), with the concomitant reduction of NAD^+^ or NADP^+^ to NADH or NADPH, respectively (Eq.1). They can also catalyze the reverse reaction [1, 2, 3, 4, 5], converting CO_2_ to formate, using NADH or NADPH as electron donors (Eq. 2).

(1) $${\text{HCOO}}^{–}\to {\mathrm{CO}}_{2}+H^{+}+{\text{2e}}^{–}$$

(2) $${\mathrm{CO}}_{2}+H^{+}+{\text{2e}}^{–}\to {\text{HCOO}}^{–}$$

The biotechnological interest in these enzymes stems from their use in both reactions: the direct reaction (formate oxidation) because of its potential in cofactor regeneration systems and the reverse (CO_2_ reduction) as a strategy for carbon capture and utilization. The ability to reduce CO_2_ under mild conditions opens the prospect of converting a greenhouse gas into a versatile chemical intermediate, amenable to downstream transformations into value‐added compounds [6, 7].

FDHs can be broadly classified into two main groups: (a) metal‐dependent enzymes, typically containing molybdenum or tungsten cofactors, and (b) metal‐independent FDHs, which rely solely on organic cofactors for catalysis [8, 9, 10]. This work focuses on the latter group, which is generally more amenable to recombinant expression and engineering and does not require complex maturation systems. However, metal‐independent FDHs typically have a lower propensity to catalyze reverse reactions.

The oxidative reaction is thermodynamically favored, proceeds efficiently over a wide pH range, and is often irreversible under physiological conditions [11, 12]. As a result, most biocatalytic applications of FDHs—and the majority of protein engineering efforts—have focused on improving oxidative activity. These include mutagenesis strategies to enhance thermostability, switch coenzyme specificity (e.g., from NAD^+^ to NADP^+^), and improve turnover numbers.

Despite these efforts, the use of native or engineered FDHs in industrial processes remains limited, due in part to moderate operational stability, suboptimal kinetics under process conditions, and the relatively high cost of enzyme production. The reductive activity of FDHs has recently gained attention owing to its relevance in sustainable bioprocesses. However, CO_2_ reduction poses additional challenges, such as its unfavorable thermodynamics and higher activation energy, which call for a deeper understanding of the structural factors that govern catalytic directionality.

Structural studies have contributed significantly to the elucidation of the function of FDH. For example, the structure of *Pseudomonas* sp. 101 FDH (*pse*FDH, PDB ID: 2NAD) revealed a catalytically preorganized active site where electrostatic interactions, particularly involving Asn146, Arg284, and His332, stabilize the formate substrate and promote hydride transfer [13]. More recently, high‐resolution structural data from *Chaetomium thermophilum* FDH (*ct*FDH, PDB: 6T8Y) [14] confirmed the role of a twisted NAD^+^ conformation and solvent‐free catalytic pocket in lowering the activation energy by facilitating access to the transition state [15]. Notably, *ct*FDH was also shown to catalyze CO_2_ reduction more efficiently than other homologs, highlighting the relevance of such structural features in both reaction directions.

In this study, we produced recombinant FDHs from four organisms—*Pseudomonas* sp. 101 (*pse*FDH), *Chaetomium thermophilum* (*ct*FDH), *Myceliophthora thermophila* (*mt*FDH), and *Ogataea parapolymorpha* (*op*FDH)—and compared their catalytic properties. We investigated the kinetic parameters of formate oxidation in the pH range. In contrast, repeated attempts to reproduce CO_2_ reduction under the conditions reported in the literature [1, 4, 5] were unsuccessful, highlighting the intrinsic limitations of wild‐type metal‐independent FDHs in catalyzing the reductive reaction.

To rationalize the observed catalytic differences, we combined molecular dynamics (MD) and quantum mechanics/molecular mechanics (QM/MM) studies. For each enzyme, we identified the transition state (TS) of the oxidative reaction and computed the associated activation free energy (*ΔG* ^*‡*^_*QM*_), which mostly correlated with the experimentally measured *k*_*ca*t_ values. We also modeled the TS of the reductive reaction and compared its barrier with that of the oxidative reaction.

The alignment between the computational and experimental data supports the hypothesis that catalytic efficiency is shaped by the extent of TS stabilization. This study provides a mechanistic basis for understanding the variable propensities of FDHs to catalyze CO_2_ reduction and may inform future engineering strategies aimed at optimizing both reaction directions.

## Results and Discussion

### Enzyme production and kinetic characterization

#### Recombinant production and purification of FDHs

**Fig. 1:**
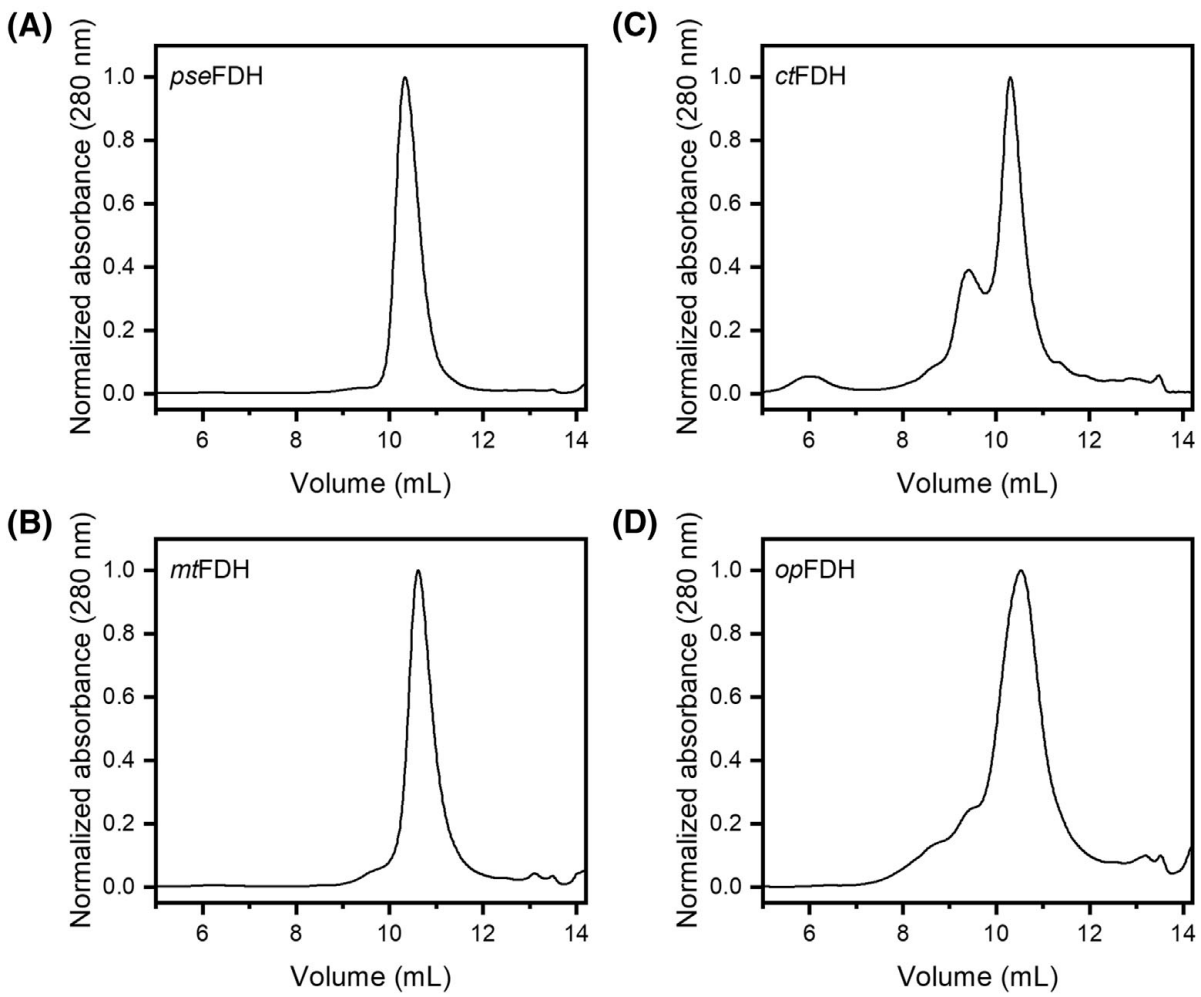
Purification and oligomeric state analysis of the four FDHs analyzed in this study. All proteins were recombinantly expressed in *E. coli* BL21 and purified by immobilized metal ion affinity chromatography (IMAC). Sample purity and quaternary structure were subsequently assessed by analytical size‐exclusion chromatography (SEC). Individual panels show (A) *pse*FDH; (B) *mt*FDH; (C) *ct*FDH; (D) *op*FDH.

All recombinant FDHs were expressed in the *E. coli* BL21 strain and IMAC‐purified to homogeneity via immobilized metal ion affinity chromatography (IMAC), as assessed by analytical size‐exclusion chromatography (SEC) (Fig 1A–D). SEC analysis displays a major elution peak for all enzymes (*pse*FDH, *mt*FDH, *ct*FDH, and *op*FDH, shown in Fig 1A–D, respectively). corresponding to molecular masses that are higher than expected for the monomeric form (theoretical monomeric weights: *pse*FDH, 44.13 kDa; *mt*FDH, 40.03 kDa; *op*FDH, 39.99 kDa; *ct*FDH, 40.84 kDa). This mass determination, achieved by comparison with a calibration curve generated using known globular standards (Figs S1, Table S1), suggests a dimeric assembly. The observed elution volumes/ retention times give rise to masses lower than those expected for dimers, likely due to the compact protein shape or to secondary interactions with the column matrix [16]. However, the dimeric assignment is highly consistent with established literature for this enzyme class [17, 18]. In addition, *ct*FDH (Fig. 1C) showed a minor peak at a lower elution volume (*V*_e_ = 9.82 mL), consistent with the formation of a higher‐order oligomeric species (likely a tetrameric assembly).

The protein amount and specific activity of each enzyme preparation measured under standard assay conditions are reported in Table S2. These data provided the basis for selecting enzyme concentrations for subsequent kinetic and structural analyses.

#### Kinetic analysis of FDH enzymes at different pH values

The kinetic parameters *K*_*m*_, *k*_*cat*_, and catalytic efficiency (*k*_*cat*_ */K*_*m*_) of the four enzymes—ctFDH, *mt*FDH, *pse*FDH, and *op*FDH—were evaluated at pH 5.0, 6.0, and 8.0 to assess the impact of pH on enzyme performance (Table 1).

**Table 1:** Kinetic parameters for formate oxidation catalyzed by FDH variants at different pH values. The table reports the Michaelis–Menten kinetic (*k*_*cat*_, *K*_*m*_ and catalytic efficiency *k*_*cat*_/*K*_*m*_) determined for the four FDH enzymes (from *Pseudomonas sp. 101*, *M. thermophila*, *C. thermophilum*, and *O. parapolymorpha*) using formate as the substrate and NAD^+^ as the cofactor. Assays were conducted at 25 °C in 0.1 m Sodium Phosphate buffer. Values are presented as the mean ± standard deviation (SD) from triplicate independent measurements (*n* = 3).

Enzyme	*K*_*m*_ (mm)	*k*_*cat*_ (s^−1^)	*k*_*cat*_ */K*_*m*_ (s^−1^ mm ^−1^)
pH 5.0			
*pse*FDH	4.65 ± 1.22	5.56 ± 0.75	1.20 ± 0.370
*mt*FDH	2.85 ± 0.76	6.62 ± 0.56	2.32 ± 0.650
*ct*FDH	6.19 ± 0,75	1.92 ± 0,22	0.31 ± 0.060
*op*FDH	7.58 ± 0.85	0.25 ± 0.01	0.03 ± 0.004
pH 6.0			
*pse*FDH	3.78 ± 0.44	4.45 ± 0.78	1.17 ± 0.24
*mt*FDH	1.88 ± 0.40	5.73 ± 1.43	3.04 ± 0.90
*ct*FDH	2.18 ± 0.61	1.22 ± 0.21	0.56 ± 0.19
*op*FDH	3.27 ± 0.49	0.13 ± 0.02	0.04 ± 0.01
pH 8.0			
*pse*FDH	5.78 ± 1.77	5.48 ± 0.10	0.95 ± 0.30
*mt*FDH	1.39 ± 0.30	5.35 ± 0.49	3.85 ± 0.96
*ct*FDH	1.41 ± 0.40	0.64 ± 0.05	0.45 ± 0.13
*op*FDH	1.74 ± 0.26	0.18 ± 0.04	0.10 ± 0.03

Among all enzymes, *mt*FDH consistently exhibited the highest catalytic efficiency, increasing from 2.32 s^−1^·mm ^−1^ at pH 5.0 to 3.04 at pH 6.0 and peaking at 3.85 at pH 8.0. This reflects a 66% increase in efficiency across the pH range, mainly driven by a high and stable *k*_*cat*_ (5.35–6.62 s^−1^) and decreasing *K*_*m*_ values (from 2.85 to 1.39 mm), indicating a slightly improved substrate affinity at higher pH.

In contrast, *ct*FDH showed moderate catalytic efficiency (0.31–0.56 s^−1^·mm ^−1^), increasing by 81% from pH 5.0 to 6.0, but slightly decreasing at pH 8.0 (0.45). This enzyme exhibited a marked improvement in substrate affinity (a 77% decrease in *K*_*m*_ from 6.19 to 1.41 mm), while its turnover number remained relatively low (1.92–0.64 s^−1^), suggesting that affinity improves with pH, but catalytic rate declines at higher pH.

*pse*FDH maintained relatively stable efficiency values across the pH range (1.20 at pH 5.0, 1.17 at pH 6.0, and 0.95 at pH 8.0), with only a 21% decrease overall. Its *k*_*cat*_ remained high (5.48–5.56 s^−1^), but the enzyme suffered from a variable substrate affinity, with *K*_*m*_ increasing significantly at alkaline pH (from 3.78 to 5.78 mm).

Finally, *op*FDH displayed the lowest catalytic efficiencies in all conditions (0.03–0.10 s^−1^·mm ^−1^), with only a minor improvement (threefold increase) from pH 5.0 to pH 8.0. Despite a noticeable improvement in affinity (*K*_*m*_ decreased from 7.58 to 1.74 mm, a 77% drop), its extremely low *k*_*cat*_ values (0.13–0.25 s^−1^) significantly limited its catalytic capacity.

Overall, *mt*FDH was the most robust and pH‐tolerant enzyme with both high catalytic turnover and affinity, whereas *op*FDH was the least efficient. The data suggest a significant pH dependence of the kinetic behavior in *ct*FDH and *op*FDH, whereas *mt*FDH and *pse*FDH maintain higher functionality across a broader pH range. Overall, the relative catalytic features of the enzymes remained consistent across the pH range tested (5.0–8.0), supporting the use of QM/MM simulations at pH 7.4 to rationalize their mechanistic differences.

On the other hand, no CO_2_‐reducing activity was detected under the conditions detailed in the *Materials and Methods* section. This lack of activity could reflect an intrinsic inability of the tested enzymes in catalyzing CO_2_ reduction and/or stem from the specific experimental setup, such as the need for an extremely high level of reduced cofactor, suboptimal environmental parameters (e.g., pH, redox potential) and low CO_2_ pressure. In fact, it must be taken into account that the equilibrium of the carbonic acid system (pK_a_ ≈ 6.1 at 25 °C) dictates the ratio of the active substrate CO_2_ and the inactive form HCO_3_^–^, substantially affecting the actual availability of the substrate. For instance at pH 7.0, the actual concentration of CO_2_ is only ≈ 1.12 mm, roughly one‐tenth of the total nominal concentration of dissolved inorganic carbon (DIC) (10 mm) (detailed calculation in *Materials and Methods*). The CO_2_ concentration tends to be even lower at pH 8.0. In general, the existence of equilibrium and its dependence on pH contribute to making accurate kinetic measurement challenging and to underestimating CO_2_ reduction activities. These limitations of the experimental system, however, underscore the value of our computational approach, which provides essential insight into the intrinsic chemical capabilities and structural features of each enzyme variant, unaffected by the bulk pH variability and substrate speciation issues.

### Computational studies of reaction mechanisms

#### Simulations of formate oxidation

The modeled reaction consists of the oxidation of formate to produce CO_2_, coupled with the simultaneous reduction of NAD^+^ to NADH. The modeling process was carried out starting with the X‐ray structures retrieved from the PDB for *pse*FDH (PDB: 2NAD) [13] and *ct*FDH (PDB: 6T8Y) [14]. Both structures were selected as the most structurally informative and computationally viable starting points for our modeling and were used directly for MD simulations. For the remaining FDHs ‐*mt*FDH and *op*FDH‐, which lack experimental structures, homology models were created using the 3D structures of FDHs from *Chaetomium thermophilum* (PDB: 6T8Y, [14]) and *Candida boidinii* (PDB: 6D4C, [19]), respectively (see ‘Materials and methods’ section). The systems obtained for all FDHs were submitted to MD simulations, followed by quantum mechanical/molecular mechanical (QM/MM) calculations on simplified models.

Specifically, the crystal structure of *pse*FDH, crystallized with azide (N_3_^−^), was used as the starting point. The azide molecule was replaced with a formate anion, representing the dissociated form of formic acid at physiological pH, while a water box spanning 10 Å from the outermost protein residue (containing 14 510 water molecules) was added. The system was submitted to 200 ns MD simulations under physiological conditions (pH 7.4, 300 K, 1 atm). This approach provided a basis for understanding the catalytic mechanism of formate dehydrogenases.

The catalytic mechanism operated by enzymes can be theoretically described using QM/MM methods, which account for the influence of residues surrounding the catalytic site. To this end, in recent years, the ONIOM hybrid approach [20, 21] has been widely used for this purpose. Previous MD studies were primarily focused just on predicting *pse*FDH active site geometries without any rationalization of catalytic performance/evaluation of their catalytic implications [22]. Calculations were performed on simplified models of enzyme–substrate complexes, which include the involved organic molecules together and the amino acids forming the catalytic site [23].

Starting from a selected frame of the final MD simulations, in which the hydride to be transferred is positioned 2.75 Å from the carbon atom of NAD^+^, a simplified model was constructed. This model included residues within a specific radius (8 Å) of the formate anion moiety. The structure was optimized using the QM/MM ONIOM method, where the ‘high level’ included NAD^+^ (reduced to the saccharide portion), the formate anion, the side chains of Arg284, His332, Asn146, Asp125, and a water molecule found near His332 during the MD simulations. Notably, His332 appears to be the closest residue to the binding site.

**Fig. 2:**
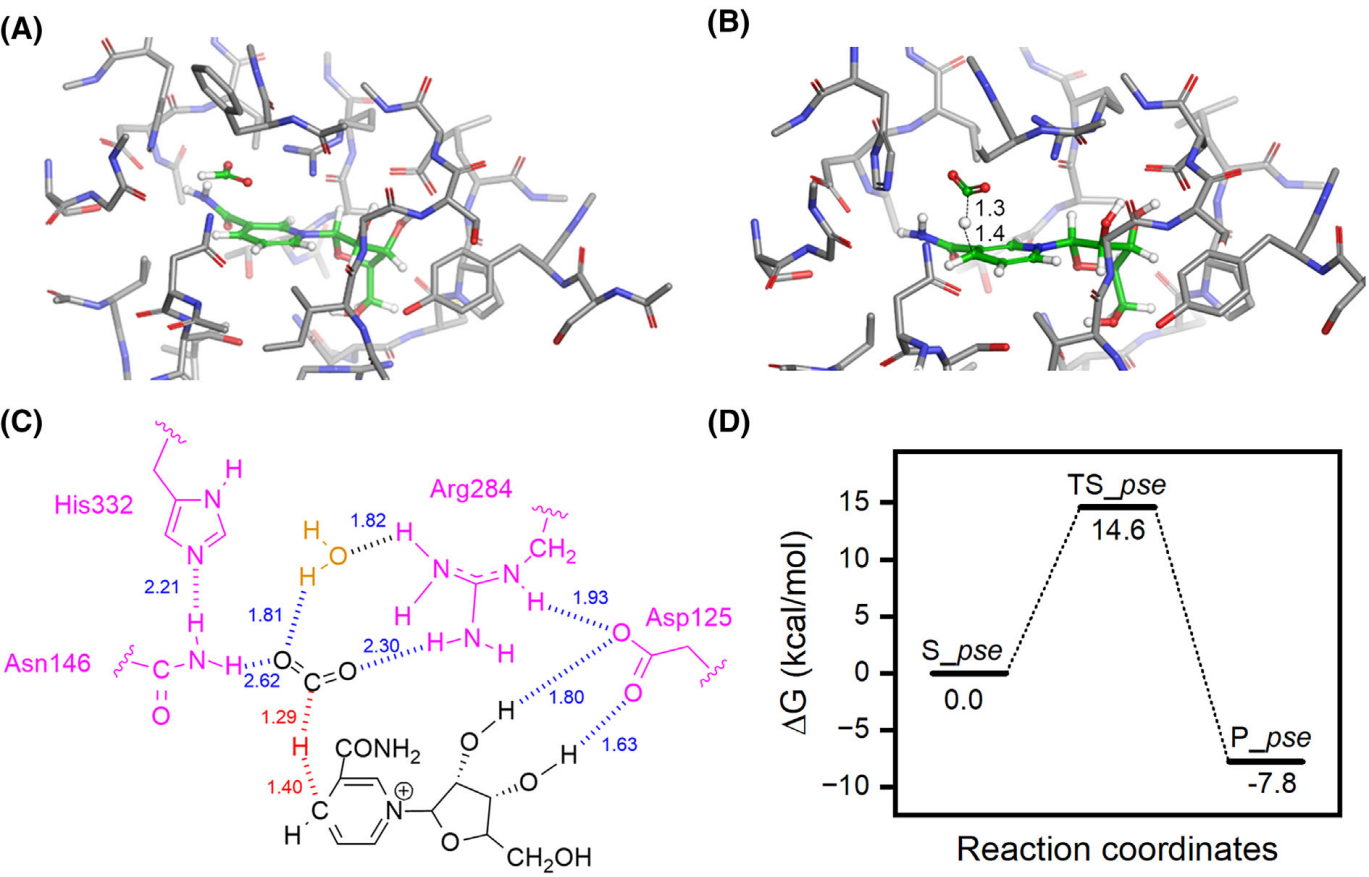
Setup of *pse*FDH‐catalyzed formate oxidation. (A) 3D representation of the optimized *pse*FDH active site with bound formate as substrate (S_*pse*). The plot was generated using pymol. (B) Corresponding 3D plot of the transition state (TS_*pse*), calculated at the B3LYP/6‐31G(D)/AM1 level. The plot was generated using pymol. In both panels, the substrates involved in the reaction are shown in green, and distances are given in Å. (C) Close‐up 2D view of TS_*pse* generated with chemdraw, highlighting key interactions involved in the hydride transfer from the formate anion to NAD^+^. Atom interactions and distances are shown in blue; forming and breaking bonds in red; residues in magenta; water molecules in orange. (D) Activation free energy (*ΔG* ^*‡*^_*QM*_) profile for the hydride transfer from the formate anion to the NAD^+^ cofactor starting from S_*pse* and giving P_*pse* as the product. Values reported on the Y‐axis represent the activation free energy (*ΔG* ^*‡*^_*QM*_), calculated via QM/MM simulations at the oniom (B3LYP/6‐31G(d):AM1) level, relative to the ground‐state reactants. The energy profile, plotted in Origin, represents the minimum energy path determined through QM/MM scans, optimizations, and vibrational frequency calculations.

For the simplified model of *pse*FDH, the transition state (TS_*pse*) leading to CO_2_ formation via hydride transfer to NAD^+^ was located. Fig. 2A,B shows 3D‐plots of the optimized simplified model with the formate anion and the corresponding transition state (TS_*pse*).

After optimizing the simplified *pse*FDH model with the formate anion, Arg284 forms hydrogen bonds with the two oxygen atoms of the formate and with a water molecule bridging to His332. Asp125 established hydrogen bonds with the hydroxyl groups at positions 2 and 3 of the ribose unit in NAD^+^.

The hydride transfer TS of *pse*FDH, located using hybrid QM/MM methods [24], highlights interactions crucial for anchoring the formate ion and preventing side reactions between the cofactor and formate. These interactions include the hydrogen bonds between Asp125 and the ribose hydroxyl groups (*d*_1_ = 1.80 Å, *d*_2_ = 1.63 Å), anchoring of the formate group to the guanidine group of Arg284 (*d* = 2.30 Å) via a bridging water molecule (*d* = 1.82 Å), and interactions with the amide group of Asn146 (*d* = 2.62 Å). The amide group of Asn146 also forms hydrogen bonds with the imidazole ring of His332 (*d* = 2.21 Å) (Fig. 2C). The catalytic water molecule critically contributes to the hydrogen‐bond network within the substrate in the active site. This network, which involves the substrate and key residues (such as Arg), is essential to stabilize the substrate in the correct geometry, thereby favoring the hydride transfer TS. The calculated free energy barrier for the *pse*FDH‐catalyzed reaction was 14.6 kcal mol^−1^, in good agreement with the literature value of 12.4 kcal mol^−1^ [24] and the experimental barrier of 16.6 kcal mol^−1^. Fig. 2D shows the free energy profile for this reaction.

A comparison with literature data [24] allows us to validate our methodology and the use of ONIOM calculations to study the FDHs derived from other microorganisms, to locate their TSs relative to hydride transfer from the formate anion to NAD^+^.

**Fig. 3:**
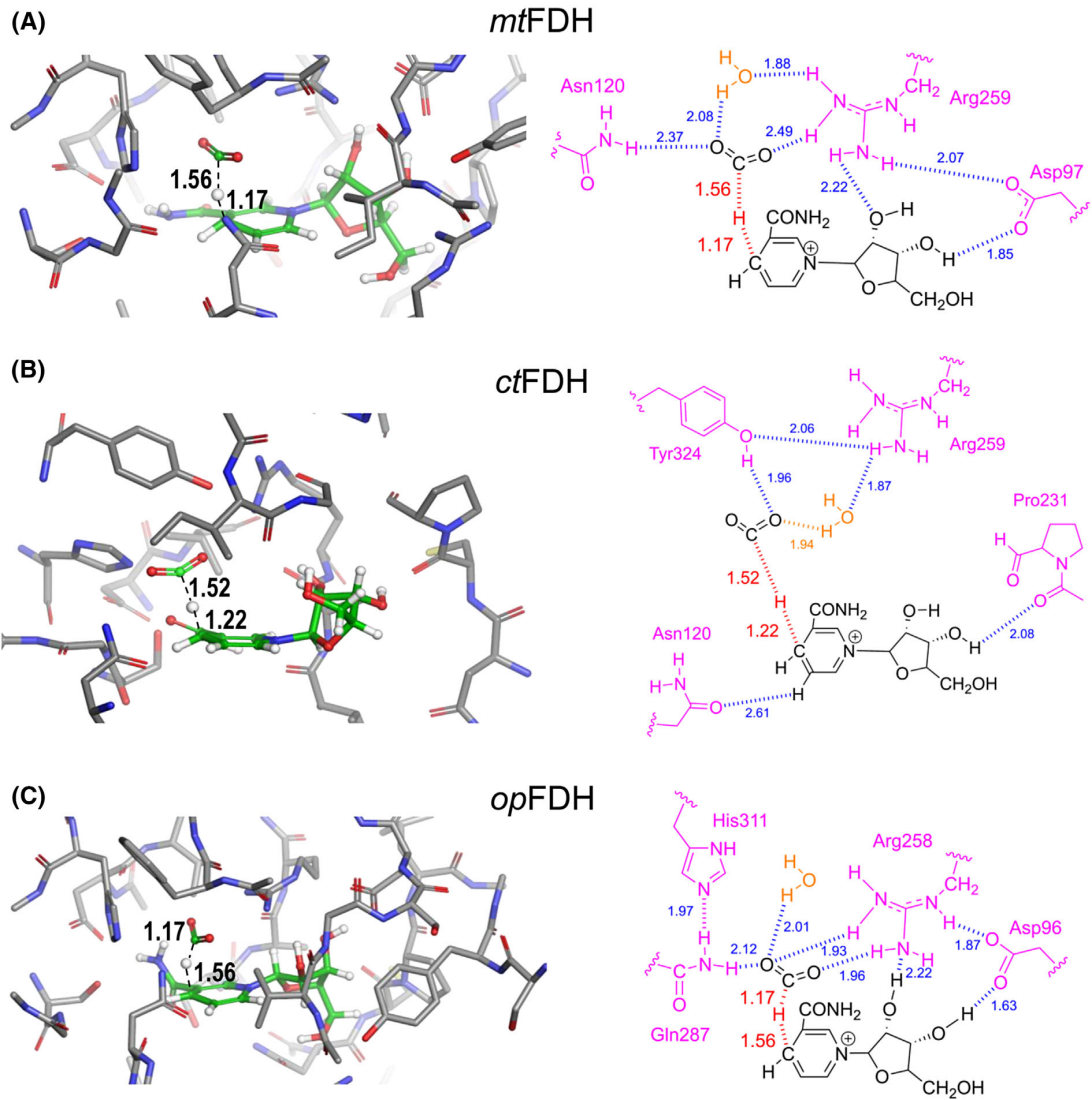
Simplified 3D models of the transition states involved in FDH‐catalyzed formate oxidation. (A) *mt*FDH, (B) *ct*FDH, and (C) *op*FDH. For each enzyme, the left panel shows the 3D structure of the catalytic region, with substrates involved in the reaction highlighted in green. Structures were optimized with Gaussian 16 and visualized using pymol. The corresponding right panel illustrates the transition‐state scheme, generated with ChemDraw, focusing on the hydride transfer from the formate anion to NAD^+^. Color coding: blue indicates atom interactions and distances in Å; red highlights forming and breaking bonds; magenta marks relevant residues; orange represents water molecules. The 3D structures and schematics represent the transition states identified along the QM/MM reaction coordinate, obtained through scan, optimization, and vibrational frequency calculations along the minimum energy path.

The same procedure was applied to all three FDHs from *M. thermophila* (*mt*FDH), *C. thermophilum* (*ct*FDH), and *O. parapolymorpha* (*op*FDH), starting with MD simulations to prepare the system, followed by ONIOM optimizations. The TSs, corresponding to the hydride transfer from formate anion to NAD^+^, resulting in CO_2_ and NADH, were located for *mt*FDH (TS_*mt*), *ct*FDH (TS_*ct*), and *op*FDH (TS_*op*) (Fig. 3A–C, respectively).

Based on the bond distances reported in Table 2, the transition‐state structures TS_*pse*, TS_*ct*, and TS_*op* appear more similar to the reactant (‘starting’) complex, as indicated by shorter dH‐C(O_2_) distances compared to dH‐C(NAD^+^). In contrast, the TS_*mt* structure more closely resembles the product complex, with dH‐C(NAD^+^) distances shorter than dH‐C(O_2_).

**Table 2:** Values of bond lengths and geometric parameters for the FDH‐catalyzed formate oxidation/NAD^+^ reduction reaction. The table reports the forming and breaking bond lengths (distances in Ångstrom, Å), the relative free energies (*ΔG* ^*‡*^_QM_ in kcal/mol), and the CĤC angle. *ΔG* ^*‡*^_QM_ values were calculated using oniom for the transition states (TS) of FDHs from *Pseudomonas* sp. 101 (TS_*pse*), *M. thermophila* (TS_*mt*), *C. thermophilum* (TS_*ct*), and *O. parapolymorpha* (TS_*op*). The CĤC angle is defined by the H atom of the formate (substrate), the C atom of the formate, and the redox‐active C4 atom of the NAD+ nicotinamide ring (H… Ĉ… C4) in the optimized TS structure of each enzyme.

Enzyme TS	dH‐C(O_2_) (Å)	dH‐C(NAD^+^) (Å)	CĤC angle (degree)	*ΔG* ^*‡*^_QM_ (kcal/mol)
TS_*pse*	1.29	1.40	158.7	14.60
TS_*mt*	1.56	1.17	160.0	16.40
TS_*ct*	1.52	1.22	167.6	20.90
TS_*op*	1.17	1.56	150.1	19.97

In TS_*mt*, the residue Asp97 forms H bonds with both the OH at position 3 of the ribose ring (*d* = 1.85 Å) and with the NH of the guanidine moiety of Arg259 (*d* = 2.07 Å). Arg259, in turn, interacts through its two NH groups with the 2‐hydroxyl group of the ribose (*d* = 2.22 Å) and with one of the O atoms of the formate ion (*d* = 2.49 Å). In this configuration, the formate ion is stabilized by two hydrogen bonds: one with a water molecule (*d* = 2.08 Å) and one with the amide group of Asn120. In analogy with the TSs of the other FDHs, TS_*ct* (Fig. 3B) features a water molecule that bridges the formate ion (*d* = 1.94 Å) to Arg259 through one of its NH groups (*d* = 1.87 Å). The substrate also forms an H bond with the OH of Tyr324 (*d* = 1.96 Å), which in turn interacts with Arg259 through a hydrogen bond (*d* = 2.06 Å). In addition, the hydroxyl group at position 2 of the ribose moiety interacts with Pro231 (*d* = 2.08 Å). Finally, TS_*op* displays essentially the same interactions network as TS_*pse* (see Fig. 3C).

**Fig. 4:**
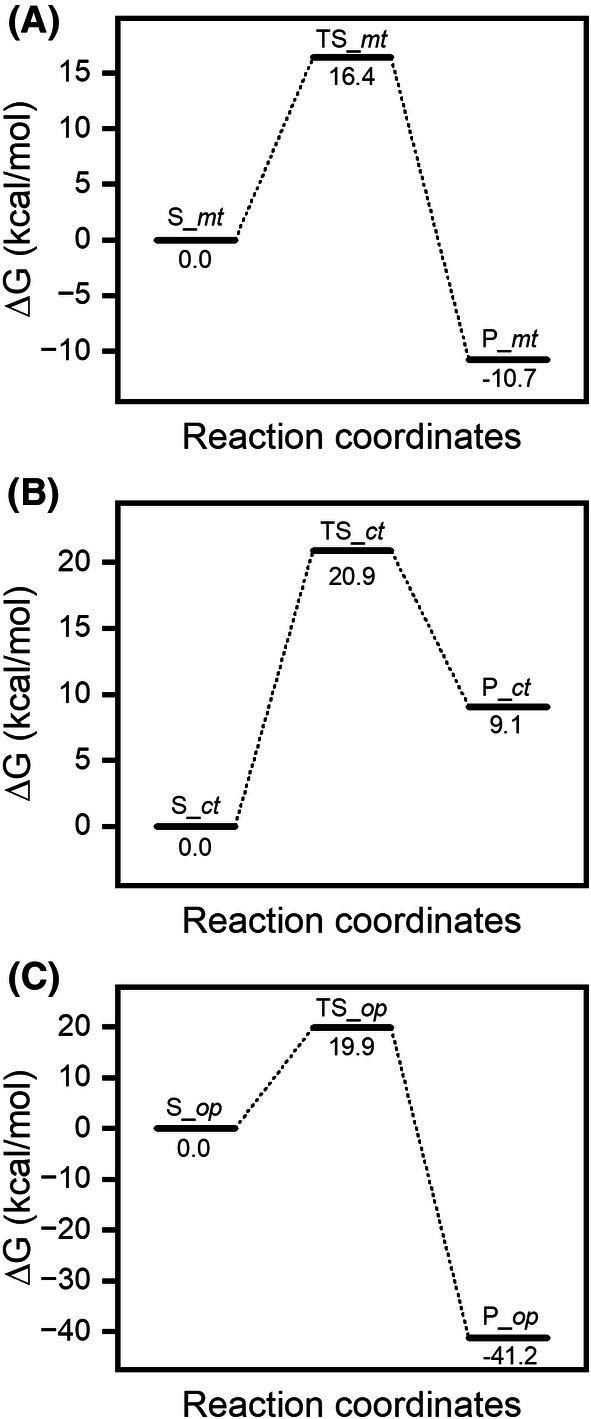
Activation free energy (*ΔG* ^*‡*^_*QM*_) profiles for FDH‐catalyzed formate oxidation coupled to NAD^+^ reduction. The reaction profiles illustrate the energy barriers for formate oxidation in different FDH variants. Values reported on the Y‐axis represent the activation free energy (*ΔG* ^*‡*^_*QM*_), calculated via QM/MM simulations at the ONIOM (B3LYP/6‐31G(d):AM1) level, relative to the ground‐state reactants (enzyme–substrate complex, S_*ct*, S_*mt*, S_*op* in A, B, and C, respectively) and giving the product of the hydride transfer (P_*ct*, P_*mt*, P_*op*). The process occurs through the corresponding transition states (TS_*ct*, TS_*mt*, TS_*op*). Energy profiles represent the minimum energy paths derived from scan, optimization, and vibrational frequency calculations, and were plotted using Origin.

Considering the relative free energy barriers (Table 2, Fig. 2D and Fig. 4), the lowest values are observed for the FDHs from *Pseudomonas* sp. 101 (TS_*pse*, *ΔG* ^*‡*^_*QM*_ = 14.60 kcal/mol) and *M. thermophila* (TS_*mt*, *ΔG* ^*‡*^_*QM*_ = 16.40 kcal/mol). For these reactions, the experimental *k*_*cat*_ values fall in the 4–7 s^−1^ range.

The highest free energy barriers were observed for *op*FDH and *ct*FDH (TS_*op*, *ΔG* ^*‡*^_*QM*_ = 19.97 kcal/mol; TS_*ct*, *ΔG* ^*‡*^_*QM*_ = 20.90 kcal/mol), which also exhibited the lowest catalytic efficiencies, with experimental *k*_*ca*t_ values of ≈ 0.2 s^−1^ and 0.6–2 s^−1^, respectively (Table 1). To further assess the reliability of our QM/MM results, we employed *k*_*cat*_ values to calculate the corresponding *ΔG* ^*‡*^ using the Eyring Equation [25, 26]. The Eyring equation, derived from transition‐state theory, provides a relationship between the rate constant (*k*) of a chemical reaction and the activation free energy (*ΔG* ^*‡*^) at a given temperature. It is expressed as (Eq. 3):

(3) $$k_{\mathrm{cat}}=\frac{k_{B}T}{h}e{\mathrm{e}}^{-\frac{\Delta G_{+}^{+}}{\mathrm{RT}}}$$

where *k*_*B*_ is the Boltzmann constant, *h* is the Planck constant, *T* is the temperature in Kelvin, *R* is the universal gas constant, and *ΔG* ^*‡*^ is the activation free energy. The *ΔG* ^*‡*^ values predicted using QM/MM simulations at pH 7.4 and 25 °C and those derived from average *k*_*cat*_ values obtained at pH 6.0 and 8.0, and 25 °C are listed in Table 3. Experimental free energy barriers (*ΔG* ^*‡*^_*exp*_) were derived from *k*_*cat*_ values obtained by averaging measurements carried out in triplicate at pH 6.0 and 8.0. This averaging provides a representative experimental benchmark across a significant portion of the enzyme activity range (see Table 1), intended to mitigate minor pH‐specific effects when comparing with theoretical values derived from a single protonation state model.

**Table 3:** Comparison of experimental and theoretical activation free energies for formate oxidation catalyzed by FDH variants. The experimental values of *k*_*cat*_ (Exp *k*_*cat*_) are the average of those obtained at pH 6.0 and 8.0 (25 °C), *ΔG* ^*‡*^_*exp*_ were calculated from Exp *k*_*cat*_ using the Eyring equation. Theoretical *ΔG* ^*‡*^_*QM*_ values were computed at pH 7.4 and 25 °C. The absolute percentage errors (APE) between average *ΔG* ^*‡*^_*exp*_ and theoretical *ΔG* ^*‡*^_*QM*_ values are also reported.

Enzyme TS	Exp. *k*_*cat*_. (s^−1^)	*ΔG* ^*‡*^_exp_ (kcal/mol)	*ΔG* ^*‡*^_QM_ (kcal/mol)	*APE* [%]
TS_*pse*	4.97 ± 0.39	16.50 ± 0.05	14.60	11.52%
TS_*mt*	5.54 ± 0.78	16.44 ± 0.08	16.40	0.24%
TS_*ct*	0.93 ± 0.11	17.49 ± 0.07	20.90	19.50%
TS_*op*	0.16 ± 0.02	18.54 ± 0.07	19.97	7.71%

The comparison between activation free energies obtained from QM/MM calculations and those derived from experimental *k*_*cat*_ values using the Eyring equation shows a generally good agreement (Table 3), supporting the reliability of the computational approach in reproducing the relative catalytic efficiency of the different enzymes. Indeed, the average absolute percentage error (APE) is below 10%, indicating that the simulations capture the main energetic trends observed experimentally. Notably, *mt*FDH exhibits excellent consistency between computed and experimental values (APE = 0.24%), while the largest deviation is observed for *ct*FDH (APE = 19.5%).

The misalignment of theoretical and experimental values may be attributed to either experimental uncertainties (e.g., catalyst concentration or assay conditions), the intrinsic approximations or limitations in the modeling, such as an inaccurate representation of the active site conformation or the computational level used (B3LYP/6‐31G(d)), and the simplifications inherent to the Eyring‐based calculation. Interestingly, the high value of Δ*G* ^*‡*^_*QM*_ suggests that *ct*FDH might favor the reverse reaction (CO_2_ reduction), prompting us to extend the mechanistic investigation in that direction.

#### Simulations of carbon dioxide reduction

**Fig. 5:**
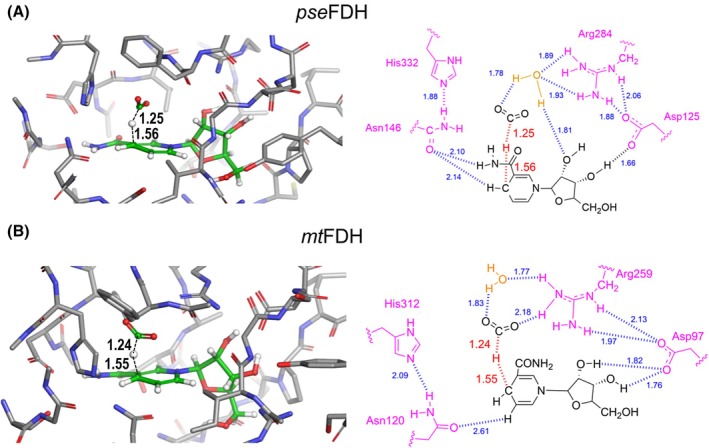
Transition states of CO_2_ reduction catalyzed by *pse*FDH and *mt*FDH. (A) *pse*FDH; (B) *mt*FDH. For each enzyme, the left panel shows the 3D structure of the transition state (TS) involved in FDH‐catalyzed CO_2_ reduction with NADH. Structures were optimized with Gaussian 16 and visualized using pymol. Substrates are highlighted in green and bond distances are given in Å. The corresponding right panel illustrates the same transition‐state schematic generated with chemdraw, focusing on the hydride transfer from NADH to CO_2_. Color coding: blue indicates atom interactions and distances; red highlights forming and breaking bonds; magenta marks relevant residues; orange represents water molecules. Both 3D structures and schematics represent the transition states identified along the QM/MM reaction coordinate, as determined by scans, optimization and vibrational frequency calculations along the minimum energy path.

**Fig. 6:**
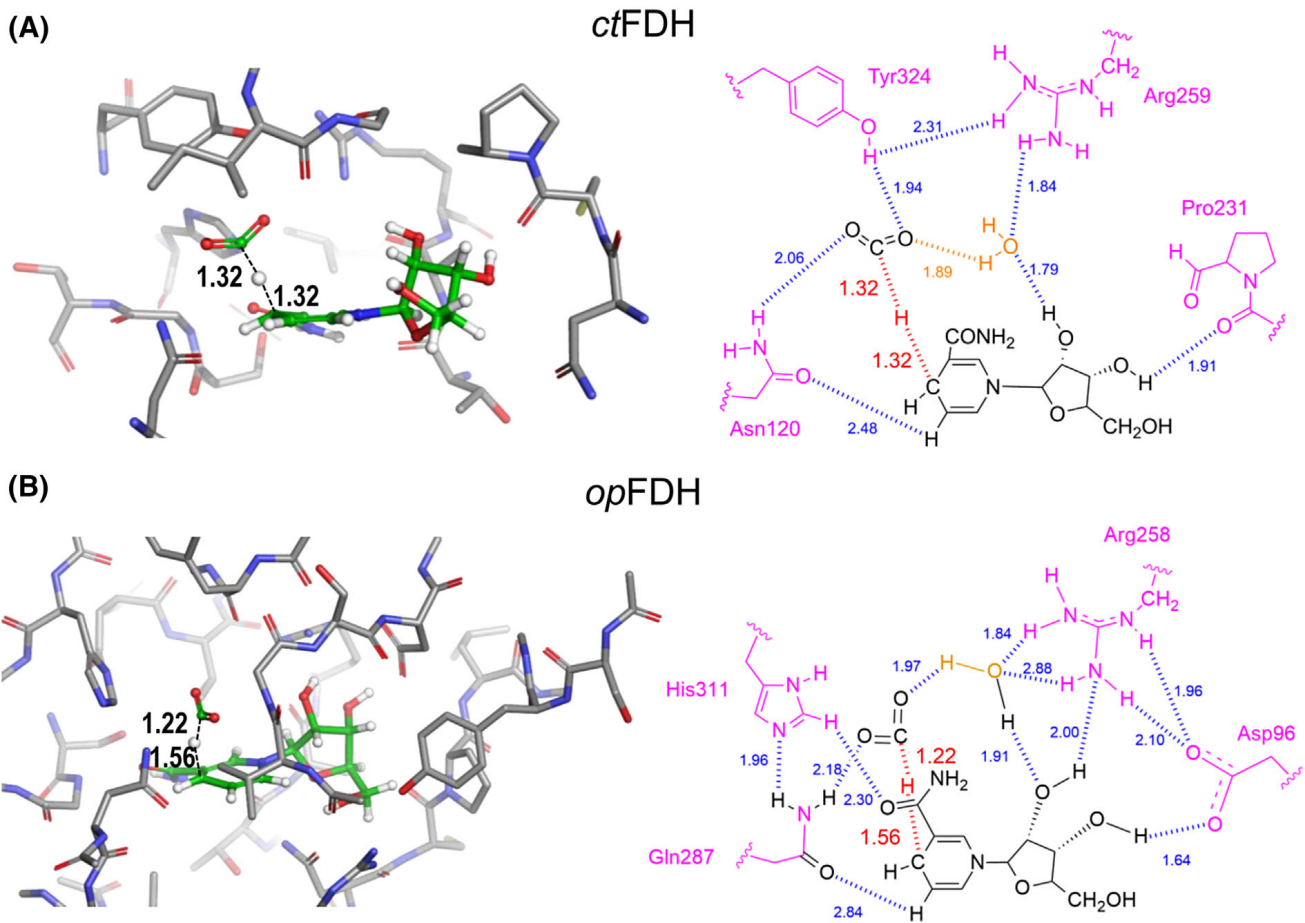
Transition states of CO_2_ reduction catalyzed by *ct*FDH and *op*FDH. (A) *ct*FDH; (B) *op*FDH. For each enzyme, the left panel shows the 3D structure of the transition state (TS) involved in FDH‐catalyzed CO_2_ reduction with NADH. Structures were optimized with Gaussian 16 and visualized using pymol. Substrates participating in the reaction are highlighted in green and bond distances are given in Å. The corresponding right panel illustrates the same transition‐state schematic generated with chemdraw, focusing on the hydride transfer from NADH to CO_2_. Color coding: blue indicates atom interactions and distances; red highlights forming and breaking bonds; magenta marks relevant residues; orange represents water molecules. The transition states were identified and validated through QM/MM reaction coordinate scans, geometry optimizations, and vibrational frequency analysis.

Besides the analysis of formate oxidation, we modeled the reverse reaction, which is the reduction of CO_2_, coupled with the simultaneous oxidation of NADH to NAD^+^. Fig. 5 and Fig. 6 show 3D representations of the TSs associated with the hydride transfer from NADH to CO_2_ in the simplified models of *pse*FDH, *mt*FDH, *ct*FDH, and *op*FDH.

In Table 4, we summarized the modeling data for the reductive reaction, implying the hydride transfer from the cofactor NADH to CO_2_. In the case of *Pseudomonas* sp. 101, compared with the TS of the direct reaction, the water molecules present in the active site showed a greater number of interactions. For the enzyme from *M. thermophila*, the interaction network is very similar to that of the direct reaction, and the O atoms of CO_2_ are anchored to the water molecule in the active site and the guanidine moiety. The most significant difference between TS_*mt* and TS_*mt*‐rev was that in the latter, Asp97 had a higher number of interactions with both the saccharide moiety and Arg259. For TS_*mt*‐rev, for the direct reaction, a dense network of H bonds was confirmed, and the number of water molecules increased to six. Finally, in TS_*op*‐rev, the network thickened with respect to the TS of the direct reaction, making this enzyme the worst catalyst for the reverse reaction.

**Table 4:** Bond distances (in Å) and transition‐state (TS) free energies (*ΔG* ^*‡*^_*QM*_, in kcal/mol) for the FDH‐catalyzed reduction of CO_2_ with NADH. *ΔG* ^*‡*^_*QM*_ values were calculated using ONIOM for the reactions catalyzed by *pse*FDH, *mt*FDH, *ct*FDH, and *op*FDH. The reported distances are dC_4_‐H(NAD), which is the distance between the C_4_ atom of NAD and the transferred hydride (representing the bond cleavage), and dH‐C(O_2_), which indicates the distance between the transferred hydride and the carbon atom of CO_2_ (representing the bond formation).

Enzyme TS	dH‐C(O_2_) (Å)	dC_4_‐H(NAD) (Å)	*ΔG* ^*‡*^_*QM*_ (kcal/mol)
TS_*pse*	1.25	1.56	26.30
TS_*mt*	1.24	1.55	22.40
TS_*ct*	1.32	1.32	10.59
TS_*op*	1.22	1.56	36.89

In the case of *Pseudomonas* sp. 101, compared with the TS of the direct reaction, the water molecules present in the active site showed a greater number of interactions. For the enzyme from *M. thermophila*, the interaction network is very similar to that of the direct reaction, and the O atoms of CO_2_ are anchored to the water molecule in the active site and the guanidine moiety. The most significant difference between TS_*mt* and TS_*mt*‐rev was that in the latter, Asp97 had a higher number of interactions with both the saccharide moiety and Arg259. For TS_*mt*‐rev, for the direct reaction, a dense network of H bonds was confirmed, and the number of water molecules increased to six. Finally, in TS_*op*‐rev, the network thickened with respect to the TS of the direct reaction, making this enzyme the worst catalyst for the reverse reaction.

From the values of the forming and breaking bond lengths reported in Table 4, the structures of TS_*pse*, TS_*mt*, and TS_*op* result to be closer to that of the final complexes, being the distances dH‐C(O_2_) shorter than the distances dC‐H(NAD), whereas in the case of TS_*ct* the two distances are very similar (dH‐C(O_2_) = 1.32 Å/dH‐C(NAD^+^) = 1.32 Å), and the structure is about halfway between reactants and products.

The lowest energy barrier in the reduction reactions was observed for *ct*FDH (*ΔG* ^*‡*^_*QM*_ = 10.59 kcal/mol), confirming our hypothesis and candidating this enzyme for possible experiments and future applications in the transformation of CO_2_ into HCO_2_.

## Conclusions

In conclusion, this study demonstrates the power of an integrated experimental and computational QM/MM approach to unravel the mechanistic details of formate oxidation for a series of FDHs, finding generally good agreement between theoretical predictions and experimental kinetics. While accurately modeling the oxidative kinetics of *ct*FDH from *Chaetomium thermophilum* presented specific challenges—highlighting the critical need to meticulously align experimental assay conditions with theoretical model parameters—our computational analysis provided a key insight. Specifically, *ct*FDH was predicted to possess the lowest activation barrier for the reverse CO_2_ reduction reaction among the enzymes investigated. This finding, coupled with its overall profile, indicates *ct*FDH as a compelling candidate for future protein engineering initiatives aimed at developing superior biocatalysts for both formate oxidation and, significantly, for efficient CO_2_ valorization.

## Materials and methods

### Biochemical and biophysical methods

#### Recombinant production of FDH in *E. coli* BL21

All FDH variants were produced containing an N‐terminal His_6_‐tag to facilitate purification. The N‐terminal placement minimizes interference with the active site environment and preserves the native catalytic properties [27, 28]. The *ct*FDH and *mtF*DH plasmids were originally described in [27, 28]; the *pse*FDH plasmid was obtained from the Addgene plasmid repository (131706) [29]; the *op*FDH plasmid was provided by Prof. Caho Gao, Shandong University, China [30]. Glycerol stocks of *E. coli* BL21 harboring FDH constructs were streaked onto LB agar plates supplemented with the appropriate antibiotics: ampicillin (25 μg/mL) for *ct*FDH, *mtF*DH, *op*FDH and streptomycin (100 μg/mL) for *pse*FDH. The plates were incubated overnight at 37 °C. Individual colonies were inoculated in 100 mL LB medium containing the corresponding antibiotics and grown overnight at 37 °C and 180 rpm. This preculture was diluted into 500 mL of production medium in 3 L flasks and incubated at 37 °C and 220 rpm. Protein expression was induced by adding isopropyl β‐d‐1‐thiogalactopyranoside to a final concentration of 1 mm when the OD_600_ reached 0.6–0.8, followed by incubation at 30 °C and 220 rpm for 30 h. Cells were harvested by centrifugation (20 000 × ***g***, 4 °C, 30 min), and the pellet was stored at − 80 °C before resuspension in 5 mL lysis buffer (300 mm NaCl, 50 mm potassium phosphate, pH 7.0, 10 mm imidazole). Lysis was performed via three rounds of sonication (1 min each, 40% power, 0 °C), and the lysate was clarified by centrifugation (20 000 × ***g***, 4 °C, 45 min).

#### Purification of recombinant FDH enzymes

Cell lysates were incubated with 1–2 mL of Ni‐NTA Sepharose 6 Fast Flow resin (GE Healthcare Technologies, Inc., Chicago, IL, USA) for IMAC, which was carried out manually in a gravity column. One mL of resin was loaded with 5 mL of cell lysate. After washing with 3 column volumes of lysis buffer, bound proteins were eluted using a 50–250 mm imidazole gradient. Eluted fractions with high dehydrogenase activity were dialyzed overnight in 20 mm phosphate buffer (pH 7.0) at 4 °C and stored at −20 °C. Sample purity was assessed by SEC using a Jasco 880‐Pu chromatograph equipped with a Yarra 3 μm SEC 3000 column (dimensions: 300 × 7.8 mm). The mobile phase consisted of 50 mm potassium phosphate buffer (pH 7.0) containing 0.15 M KCl, flowing at a rate of 0.3 mL/min. The absorbance signal was recorded at a wavelength of 280 nm.

System calibration was performed using a set of proteins with known hydrodynamic radii (MS2 bacteriophage capsid, 2560 kDa; bovine serum albumin, dimeric form, 132 kDa; bovine serum albumin, monomeric form, 64.7 kDa; ovalbumin, 42.9 Da; myoglobin, 17.2 kDa; cytochrome C, 12.4 kDa; phenylalanine, 165 Da). Protein solutions were prepared at a concentration of 1 mg/mL, 20 μL of which were injected into the column.

#### Protein quantification

Protein concentrations were determined using UV absorption at 278 nm and the Beer–Lambert law, with extinction coefficients calculated using ProtParam. Absorbance was measured using a Jasco V‐730 spectrophotometer.

#### Assays of formate oxidation and NaHCO_3_ reduction

The oxidative activity of the FDH enzymes was determined using sodium formate as substrate and monitoring the NAD^+^ reduction to NADH at 340 nm (ɛ = 6.22 mm ^−1^ cm^−1^) over time by a double‐beam UV–Vis spectrophotometer (Jasco V‐650) at 25 °C in quartz cuvettes with a 1 cm path length. NADH formation rate, measured as Abs/min within the linear range of the assay, was used to determine the relative catalytic activity of the enzymes.

The proper amount of enzyme in 20 μL was added to the reaction mixture containing 0.3 m sodium formate, 0.1 mm NAD^+^ in 0.1 m sodium phosphate buffer (pH 7.0), in a final volume of 1 mL.

When testing formate synthesis, the same reaction conditions were used, except that a 10 mm sodium hydrogen carbonate solution and 1 mm NADH were used instead of sodium formate and NAD^+^, respectively. To accurately determine the concentration of the active substrate (CO_2_) used in the CO_2_ reduction assays, we performed calculations based on the carbonic acid equilibrium. The reaction mixture contained 10 mm sodium bicarbonate (HCO_3_^−^) at a pH of 7.0, resulting in a total DIC of 10 mm. Using the Henderson–Hasselbalch equation and the apparent pK_a1_ = 6.1 (at 25 °C) for the CO_2_ / HCO_3_^−^ system:

(4) $$\mathrm{pH}=pK_{a_{1}}+\log\left(\frac{\left[\mathrm{HC}O_{3}^{-}\right]}{\left[CO_{2}\right]}\right)$$

(5) $$7.0=6.1+\log\left(\frac{\left[\mathrm{HC}O_{3}^{-}\right]}{\left[CO_{2}\right]}\right)$$

This calculation yields an approximate ratio of [HCO_3_^−^]/[CO_2_] ≈ 7.94. Given that [HCO_3_^−^] + [CO_2_] = 10 mm, the estimated concentration of the active substrate, dissolved CO_2_, present in the solution is approximately 1.12 mm.

The NADH formation/depletion rate, measured as Abs/min within the linear range of the assay, was used to determine the relative catalytic activity of the enzymes, applying the molar extinction coefficient of NADH (ɛ = 6.22 mm ^−1^ cm^−1^). Each assay was performed in triplicates.

#### Determination of kinetic parameters at various pH values

Kinetic parameters (*K*_*m*_, *k*_*cat*_ and *V*_*max*_) were determined for the oxidation of formate under saturated NAD^+^ concentration (0.1 mm) and varying sodium formate concentrations (0.2–150 mm). All measurements were performed at 25 °C using 0.1 m sodium phosphate buffer to determine kinetic parameters at pH 6.0 and 8.0, and 0.1 m sodium acetate buffer to carry out assays at pH 5.0. The reactions were initiated by addition of the enzyme, and each condition was tested in triplicate. Kinetic data were analyzed by fitting the initial rate measurements to the Michaelis–Menten equation.

### Computational methods

#### Preparation of X‐ray structures

The X‐ray 3D structures of *pse*FDH and *ct*FDH were retrieved from the PDB (PDB IDs: 2NAD [13] and 6T8Y [14], respectively). Both structures were used directly for MD simulations.

The 3D structure of *pse*FDH is available in complex with azide and NAD^+^ but not with formate. To generate a plausible enzyme–substrate complex, the azide moiety was manually replaced with a formate ion (HCOO^−^) using Maestro Build tool.

The *ct*FDH structure shows NADH bound to the catalytic site, while the formate ion is located in a secondary binding site distinct from the active center. To model a canonical Michaelis–Menten complex, we performed a structural alignment with *pse*FDH (PDB 2NAD), allowing the formate ion—positioned in the catalytic site in *pse*FDH—to be superimposed onto the *ct*FDH active site. This hybrid approach exploited the conserved geometric features of NAD^+^‐dependent FDHs across species. The resulting complexes were processed using the ‘Protein Preparation Wizard’ (Maestro release 2024–2, Schrödinger) through the following steps: (1) assignment of protonation states at pH 7.4; (2) structural validation and resolution of steric clashes; (3) application of the OPLS4 force field. Systems were solvated in TIP3P water boxes, energy‐minimized, and subjected to 200 ns MD simulations using Desmond (release 2024–2, Schrödinger, LLC, New York, NY, USA). MD simulations were performed simulating a physiological pH environment, corresponding to approximately pH 7.4 for system neutralization. The protonation states of all titratable residues were determined prior to MD simulations to ensure a chemically relevant/suitable representation of the active site. In this context, the histidine tag is not included since far from the catalytic site and not affecting the transition state. Additionally, the Maestro Epik module predicted for the catalytic His the same protonation state at both pH 5 and pH 8.

During MD simulations, the formate dissociated from the active site in both *pse*FDH and *ct*FDH systems before completion of the trajectories. To exclude potential artifacts related to the OPLS4 force field used in the Desmond simulations, we performed additional 250 ns MD simulations on the *pse*FDH/formate complex using the ff19SB [31] and GAFF2 [32] force fields for the protein and ligand, respectively. These control simulations yielded the same results (Fig. S2). In contrast, the NAD^+^ cofactor remained stably bound throughout the trajectories. Furthermore, root mean square deviation (RMSD) analyses of the Cα atoms indicated that both enzymes rapidly reached conformational equilibrium (Fig. S3). This transient binding behavior aligns with the weak interactions typically exhibited by small, highly mobile substrates within enzymatic binding pockets.

To account for this dynamic variability, we systematically analyzed MD trajectories to identify snapshots in which the distance between the H atom of the formate ion and the redox‐active C4 atom of the NAD^+^ nicotinamide ring was minimized. These geometrically favorable configurations, extracted from each system, were used as starting structures for hybrid quantum QM/MM calculations using the oniom methodology.

#### Preparation of homology models

Due to the lack of experimental structures for *mt*FDH (UniProt G2QB71) and *op*FDH (UniProt W1Q801), we generated homology models using the swiss‐model server [33]. This platform identified suitable template structures through automated alignment. Specifically, *mtF*DH was modeled using the structure of the homologous enzyme from *Chaetomium thermophilum* (PDB 6T8Y [14]), due to its high sequence identity (92.16%), strong similarity (95.14%), and minimal alignment gaps (0.81%). *op*FDH was modeled using the *Candida boidinii* FDH X‐ray structure (PDB 6D4C [19]), based on its superior sequence identity (83.61%), similarity (92.08%), low alignment gaps (1.64%) based on BLOSUM62 matrix scoring and high structural resolution.

The initial homology models were aligned to the NAD^+^/azide‐bound structures of the respective templates (PDB 6T8Y for *mt*FDH; PDB 6D4C for *op*FDH). Azide was manually inserted into each model and subsequently converted into a formate ion using the Maestro Build tool. Both enzyme–substrate complex models were then refined by following the same protocol described for enzymes with available crystal structures, including system preparation and MD simulations. RMSD plots of Cα atoms are reported in Fig. S3. Finally, structures for QM/MM calculations were chosen from the trajectory frames exhibiting the shortest distance between the formate H atom and the redox‐active C_4_ atom of the NAD^+^ nicotinamide moiety.

#### oniom calculations

Simplified models were extracted from selected MD simulations frames by retaining all atoms within a 4 Å radius centered on the NAD^+^ cofactor, including a water molecule located at the catalytic site. All calculations were carried out using the Gaussian16 program package [34]. Prior to the QM/MM calculations, the protonation states of all titratable residues were determined using the Epik module of Maestro at physiological pH (∼ 7.4). Crucially, a subsequent analysis confirmed that the protonation state of the key catalytic residues within the active site remained unchanged when calculated at pH 5.0 and pH 8.0, indicating the stability of the active site model across the experimental pH range.

Geometry optimizations were performed using the oniom hybrid method, applying density functional theory (DFT) at the B3LYP/6‐31G(d) level to the ‘high layer’ [35, 36].

The ‘low layer’, which was held fixed during optimization, was treated with the semiempirical AM1 method [37].

The high‐level layer included the truncated NAD^+^ molecule (limited to the ribose moiety), the formate anion, a catalytic water molecule, and the side chains of four key residues: Arg284, His332, Asn146, and Asp125. The low‐level layer enclosed the surrounding atoms within the model, with hydrogen atoms used to cap the truncated bonds at the QM/MM boundary.

Vibrational frequency analyses were performed at the same level of theory to verify the nature of the stationary points, distinguishing energy minima from transition states by the presence of a single imaginary frequency corresponding to bond formation.

## Author contributions

LL designed and performed experiments, analyzed data, wrote, and revised the manuscript; MG performed experiments; EL performed experiments; EMAF designed and performed experiments, analyzed data; GG performed experiments, analyzed data, and wrote the manuscript; MAC performed experiments and analyzed data; BB analyzed data, critically reviewed and advised on the final revisions of the manuscript; SB designed experiments, supervised research, analyzed data, wrote, and revised the manuscript; FS designed experiments, analyzed data, supervised research, and revised the manuscript.

## Conflicts of interest

The authors declare no conflicts of interest.

## Supporting information


**Fig. S1** SEC calibration plot.
**Fig. S2**. Time evolution of the formate/NAD^+^ interatomic distance during MD control simulation.
**Fig. S3**. Cα atoms root mean square deviation (RMSD) plot for FDH variants.
**Table S1**. Estimated molecular masses (MW) of FDH enzymes determined by SEC analysis.
**Table S2**. Purification yield and specific activity of FDH variants.

## Data Availability

Data supporting the results included in this article are available upon request to the corresponding authors.
