# Supplementary material for: Insights into the catalytic mechanism of formate dehydrogenases from different microbial sources
Source: FEBS J. 2026 Mar 5;293(15):4645–59. doi: 10.1111/febs.70477 (PMC13440586; doi:10.1111/febs.70477)
Supplement: Supplementary file 1 [file FEBS-293-4645-s001.pdf]

Insights into the catalytic mechanism of formate dehydrogenases from different microbial sources

Laura Legnani[1], Marco Gargiulo[2], Elia Lio[2], Enrico Mario Alessandro Fassi[3], Giovanni Grazioso[3], Maria Assunta Chiacchio[4], Barış Binay[5, 6], Stefania Brocca[1],* Francesco Secundo[2*]

Supplementary Figures and Tables

Figure S1. SEC calibration Plot

Figure S2. Time evolution of the Formate/NAD$^+$ interatomic distance during MD control simulation.

Figure S3. Cα Atoms Root Mean Square Deviation (RMSD) plot for FDH variants.

Table S1. Estimated molecular masses (MW) of FDH enzymes determined by SEC analysis.

Table S2. Purification yield and specific activity of FDH variants.

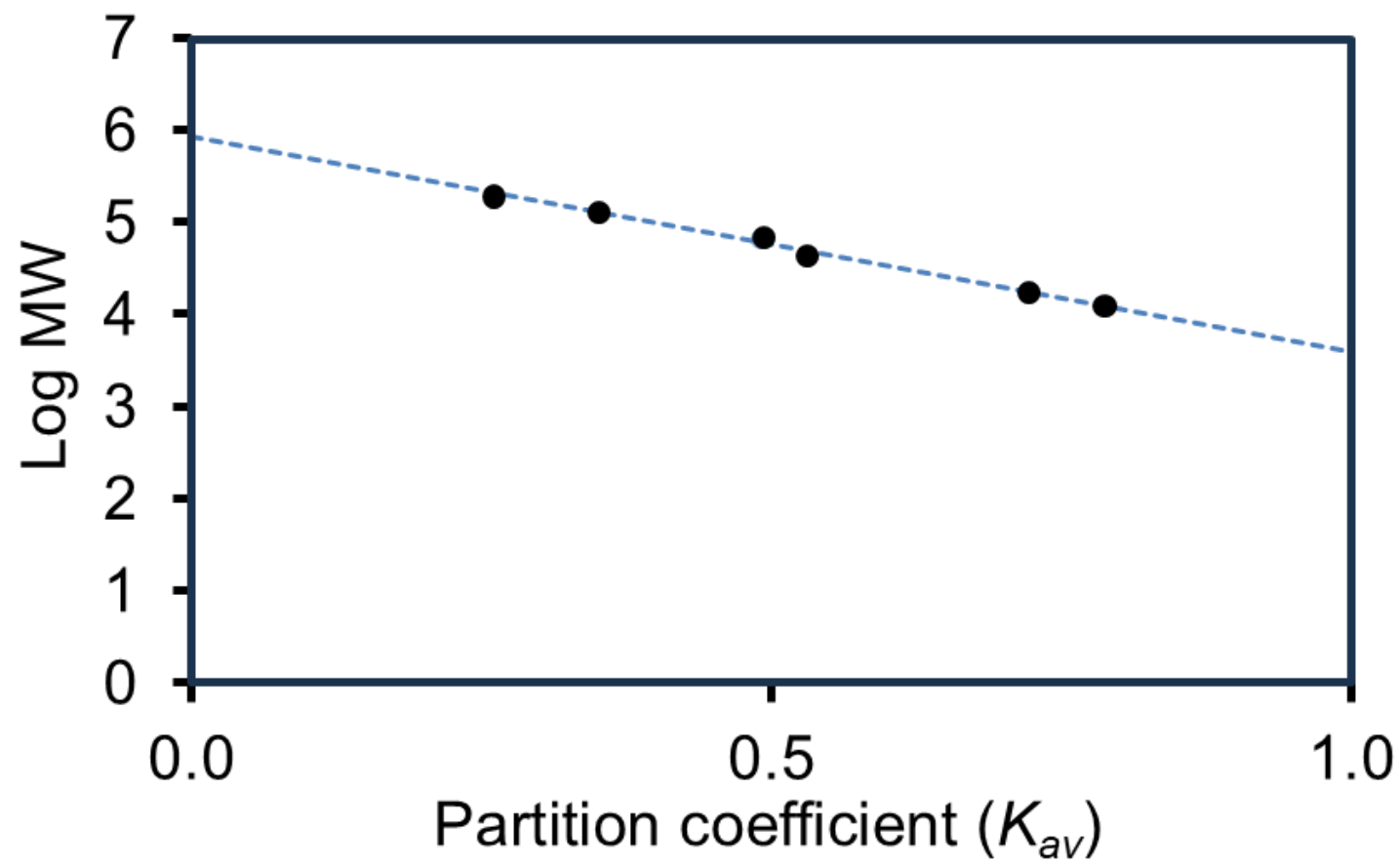

Fig. S1. SEC calibration plot. Calibration plot (slope =-2.33, intercept= 5.92) of the Yarra 3μm SEC-3000 chromatographic column, obtained using standard proteins: bovine serum albumin (monomer 66.5 kDa, elution volume (V_e) = 10.4 mL; dimer 133.0 kDa, V_e = 9.41 mL; trimer 199.5 kDa, V_e = 8.77 mL), ovalbumin (43 kDa, V_e = 10.68 mL), myoglobin (17.6 kDa; V_e = 12.03 mL), and cytochrome C (12.4 kDa; V_e = 12.49 mL). The partition coefficient (K_{av}) was calculated as (V_e-V_o)/(V_t-V_o), where the V_o and V_t represent the void volume and total column volumes, determined from the elution of MS2 phage capsid (2,560 kDa) and tyrosine (182 Da), respectively.

Table S1. Estimated molecular masses (MW) of FDH enzymes determined by SEC analysis with Yarra 3μm SEC-3000 column.

Elution volumes (V_e) and corresponding apparent MW were calculated from the calibration curve shown in **Figure S1** (y = –2.326x + 5.9204). Molecular masses were estimated from the retention volumes using the partition coefficient $K_{av} = (V_e - V_0)/(V_t - V_0)$, where $V_0$and V_t correspond to the void and total column volumes, respectively.

Protein	V_e (mL)[a]	Molecular mass (kDa)
*pse*FDH	10.325	91.0
*mt*FDH	10.625	59.0
*ct*FDH	10.867	42.7
*op*FDH	10.592	61.5

(Theoretical MW of FDHs calculated on the base of their amino acid sequences: *pse*FDH: 44.13 kDa, *mt*FDH: 40.03 kDa, *ct*FDH: 40.84 kDa, *op*FDH: 39.99 kDa).[a] Elution volumes (V_e) refer to the main peak of chromatograms shown in Figure 1.

Table S2. **Purification yield and specific activity of FDH variants.** The table reports the total protein yield, total activity, and specific activity obtained after the final purification step. FDH activity was assayed at pH 7.0 and 25 °C (using formate and NAD^+).

Enzyme	Total protein (mg)	Total activity (mU)	Specific activity [a] (mU/mg)
*ct*FDH	38.7	924.9	23.9
*mt*FDH	30.3	1715.0	56.6
*pse*FDH	9.0	141.3	15.7
*op*FDH	16.5	31.3	1.9

[a]Specific activity is expressed in milliUnits per milligram of protein, where one enzymatic unit (U) is defined as the amount of enzyme required to catalyze the reduction of 1 µmol of NAD^+ per minute.

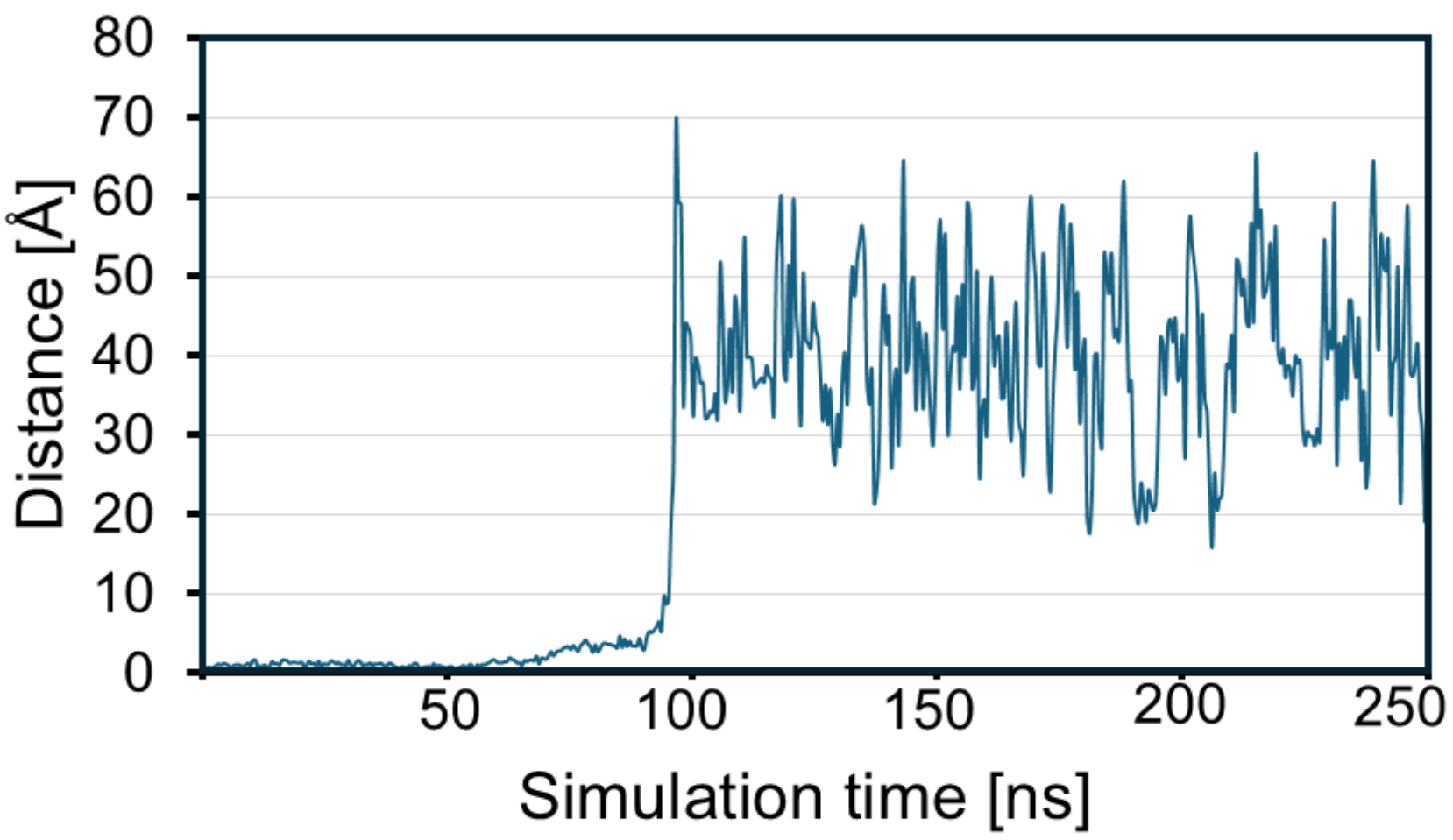

Figure S2. Formate/NAD(H) binding stability from MD simulation. Trace showing the time evolution of the interatomic distance (in Å) between the hydrogen (H) atom of the formate substrate and the redox-active carbon (C4) atom of the NAD^+ nicotinamide ring. Data were obtained from a 250 ns MD simulation of the *pse*FDH (2NAD) complex, performed using the AMBER ff19SB (for the protein) and GAFF2 (for the ligand) force fields. The sharp increase in distance at ~94 ns confirms the dissociation of formate from the active site, demonstrating that the transient binding behavior is an intrinsic property of the enzyme-substrate complex rather than a force field artifact.

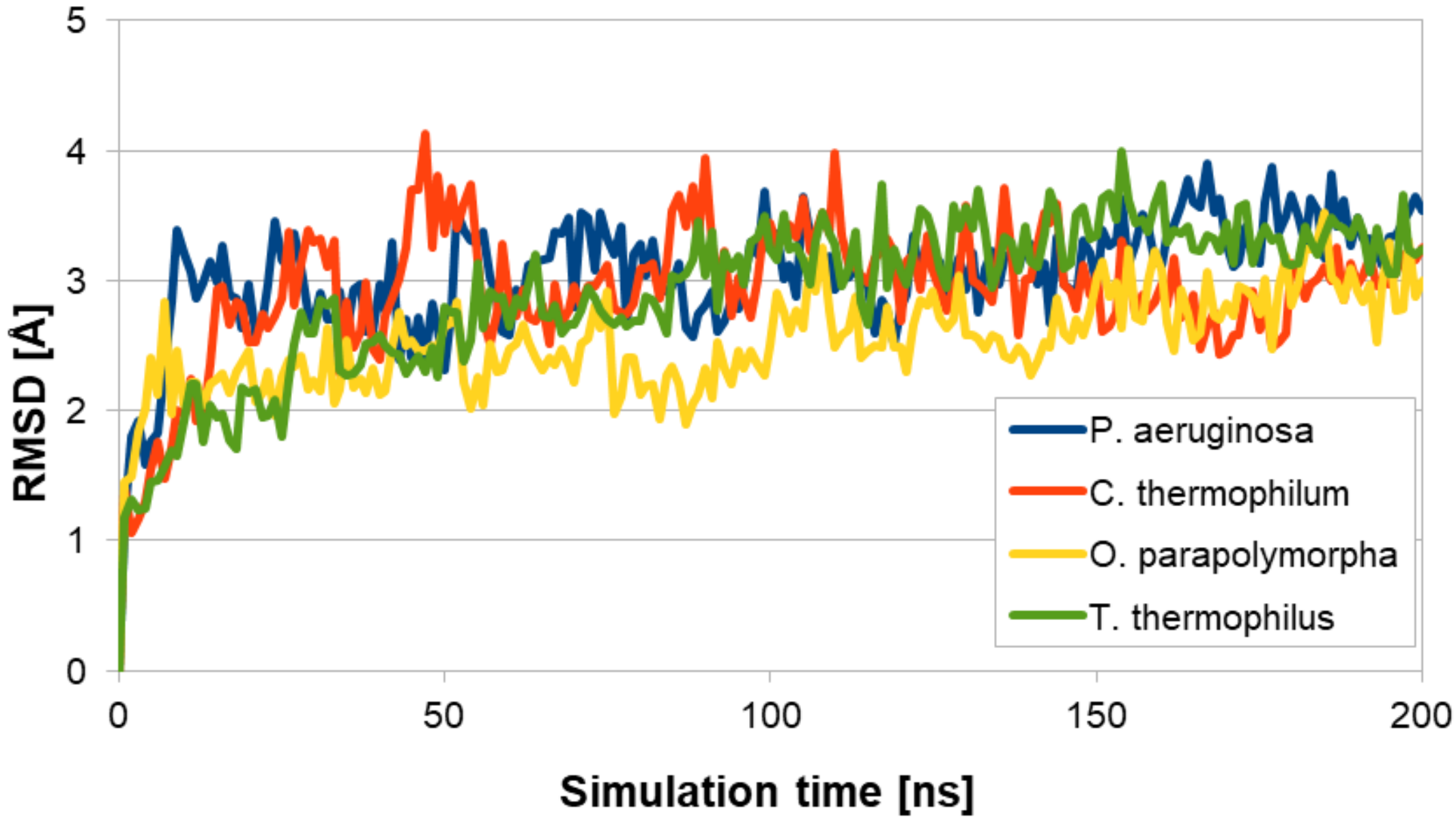

Figure S3. Cα atoms Root Mean Square Deviation (RMSD) plot for FDH variants. RMSD calculated for the Cα backbone atoms from MD simulations (200 ns each) accomplished on enzymes from *Pseudomonas sp.* 101 (*pse*FDH), *Myceliophthora thermophila* (*mt*FDH), *Chaetomium thermophilum* (*ct*FDH), and *Ogataea parapolymorpha* (o*p*FDH). The plots confirm the rapid attainment of conformational equilibrium.
